# Marital status and risk of cardiovascular disease – a multi-analyst study in epidemiology

**DOI:** 10.1007/s10654-025-01235-8

**Published:** 2025-05-05

**Authors:** Bernd Kowall, Linda Juel Ahrenfeldt, Jale Basten, Heiko Becher, Tilman Brand, Julia Braun, Swaantje Casjens, Heiner Claessen, Robin Denz, Hans H. Diebner, Sophie Diexer, Nora Eisemann, Eva Furrer, Wolfgang Galetzka, Carolin Girschik, André Karch, Rafael Mikolajczyk, Manuela Peters, Susanne Rospleszcz, Viktoria Rücker, Andreas Stang, Susanne Stolpe, Katherine J. Taylor, Nina Timmesfeld, Marianne Tokic, Hajo Zeeb, Gabriele Berg-Beckhoff, Thomas Behrens, Till Ittermann, Nicole Rübsamen

**Affiliations:** 1 Institute of Medical Informatics, Biometry and Epidemiology (IMIBE), University Hospital of Essen, Hufelandstraße 55, 45147 Essen, Germany; 2 Research Unit for General Practice, Department of Public Health, University of Southern Denmark, Esbjerg-Odense, Denmark; 3 Department of Medical Informatics, Biometry and Epidemiology, Ruhr-University Bochum, Bochum, Germany; 4 Institute of Global Health, Heidelberg University Hospital, Heidelberg, Baden-Württemberg, Germany; 5 Leibniz Institute for Prevention Research and Epidemiology – BIPS, Bremen, Germany; 6 Departments of Biostatistics and Epidemiology, Epidemiology, Biostatistics and Prevention Institute, University of Zurich, Hirschengraben 84, Zurich, CH-8001 Switzerland; 7 Institute for Prevention and Occupational Medicine of the German Social Accident Insurance, Institute of the Ruhr University Bochum (IPA), Bochum, Germany; 8 Institute of Health Services Research and Health Economics, German Diabetes Center, Leibniz Center for Diabetes Research at Heinrich Heine University Düsseldorf, Düsseldorf, Germany; 9 Institute for Medical Epidemiology, Biometrics, and Informatics, Interdisciplinary Centre for Health Sciences, Medical Faculty of the Martin, Luther University Halle-Wittenberg, Halle (Saale), Germany; 10 Institute of Social Medicine and Epidemiology, University of Lübeck, Lübeck, Germany; 11 Center for Reproducible Science, University of Zurich, Zurich, Switzerland; 12 Institute of Epidemiology and Social Medicine, University of Münster, Münster, Germany; 13 Department of Diagnostic and Interventional Radiology, Medical Center, Faculty of Medicine, University of Freiburg, Freiburg, Germany; 14 Institute of Epidemiology, Helmholtz Munich, Neuherberg, Germany; 15 Institute of Clinical Epidemiology and Biometry, Julius-Maximilians-Universität Würzburg, Würzburg, Germany; 16 School of Public Health, Department of Epidemiology, Boston University, Boston, USA; 17 Institute of Medical Biostatistics, Epidemiology, and Informatics, University Medical Centre, Mainz, Germany; 18 Health Sciences Bremen, University of Bremen, Bremen, Germany; 19 Department of Public Health, Research Unit for Health Promotion, University of Southern Denmark, Esbjerg, Denmark; 20 Institute for Community Medicine, University Medicine Greifswald, 17475 Greifswald, Germany

**Keywords:** Multi-analyst study, Multiverse analysis, Many analysts, Arbitrary choices, Researcher degrees of freedom

## Abstract

In multi-analyst studies, several analysts use the same data to independently investigate identical research questions. Multi-analyst studies have been conducted mainly in psychology, social sciences, and neuroscience, but rarely in epidemiology. Sixteen analyst groups (24 researchers) with backgrounds mainly in statistics, mathematics, and epidemiology were asked to independently perform an analysis on the influence of marital status (never married versus cohabiting married) on cardiovascular outcomes. They were asked to use data from the Survey of Health, Ageing and Retirement in Europe (SHARE), a panel study of 140,000 persons aged 50 years and above from 28 European countries and Israel, and to provide an effect estimate, a comment on their results, and the full syntax of their analyses. In additional analyses beyond the multi-analyst approach, one group selected an exemplary regression model and varied definitions of exposure and outcome and the confounder adjustment set. Each analysis was unique. The size of the 16 datasets used for the analyses ranged from 15,592 to 336,914 observations. The effect estimates (odds ratios, hazard ratios, or relative risks) ranged from 0.72 to 1.02 (reference: cohabiting married) in strictly or partly cross-sectional analyses and from 0.95 to 1.31 in strictly longitudinal analyses. The choice of regression models, adjustment sets for confounding, and variations in the precise definition of exposure and outcome, all had only small effects on the effect estimates. The range of results was mainly due to differences from cross-sectional versus longitudinal analyses rather than to single analytical decisions each of which had less influence.

**Supplementary Information:**

The online version contains supplementary material available at 10.1007/s10654-025-01235-8.

## Introduction

In epidemiology, results for the same research question often vary widely for several reasons: Populations may differ in mean and distribution of individuals’ characteristics. Study samples are subject to sampling error. Measurements of variables vary. Also, even for the same research question and identical data, researchers have a wide range of analytical choices [1]. Hoffmann et al. distinguish between sampling uncertainty, measurement uncertainty and the multiplicity of possible analysis strategies [1]. To date, the latter point of view has not received much attention in epidemiology.

Various terms have been coined for strategies to address heterogeneity in analysis strategies such as “vibration of effects”, “multiverse analysis”, and “specification curve analysis” [2–4]. Vibration of effects refers to the variation in effect estimates when multiple analytical modelling approaches are applied [2]. In multiverse analyses, a large set of processed data sets (“a data multiverse”) is constructed from given raw data rather than constructing a single processed data set [3]. Similarly, a “model multiverse” can be constructed that includes all justifiable statistical models [3]. Specification curve analysis is a sophisticated procedure in which the dispersion of the estimated effect sizes is graphically represented across a large number of analytical choices [4]. Other terms are “non-standard error” in the evidence generating process as opposed to the standard error in the data generating process, and “researcher degrees of freedom” [5, 6]. The latter are legitimate micro-decisions and are usually not clear and definite errors. Researcher degrees of freedom are distinct from p-hacking which means deliberately making analytical choices which lead to statistically significant results. P-hacking and searching for desired results can be avoided by preregistration of studies, by recording the analysis strategy in an analysis protocol before receiving the data, and – in randomized trials – by hiding the assignment of study participants from the researchers who analyze the data. However, preregistration does not prevent results from depending on subjective, but defensible strategies of analysis [7].

So far, many multi-analyst studies have been carried out in neurosciences, psychology, social sciences, and economics [5, 6, 8–17]. Multi-analyst studies have often produced a wide range of results, frequently including a change in the direction of the central result. A multi-analyst study, which was probably the first and which attracted a lot of attention, involved 29 groups of analysts and investigated whether referees give red cards more often to footballers with dark skin; the odds ratios ranged from 0.89 to 2.93 [11]. In an econometric study, seven researchers were given two research questions [6]. The results for the first question, on compulsory schooling and teenage pregnancy, varied widely, whereas the results for the second question, on health insurance and entrepreneurship, were much more consistent. Thus, in multi-analyst studies in the literature, large variations in results have been observed, but there are also exceptions to the rule.

In the meantime, guidelines for multi-analyst studies have been published [18]. Wagenmakers et al. recommend further multi-analyst studies to test the proposition that researcher degrees of freedom produce a wide range of results [19]. We conducted a multi-analyst epidemiological study using data from the Survey of Health, Ageing and Retirement in Europe (SHARE), a large European social science panel study [20, 21]. The main interest and the rationale of our study was to examine how researcher degrees of freedom affect estimates in epidemiology. In addition to the multi-analyst study, one team used an example dataset to look at how specific changes in the analysis affect the effect estimates when everything else is held constant. These specific changes included comparing cross-sectional and longitudinal analyses, varying the definition of exposures and outcomes, and adjusting for confounders.

## Methods

The first author and a co-worker from the Institute of Medical Informatics, Biometry and Epidemiology at the University Hospital in Essen contacted 28 researchers who held professorships in biometry, epidemiology, or public health (25 from Germany, 1 from Switzerland, 1 from Austria, 1 from Denmark) and invited them to participate in the multi-analysist study themselves or to nominate researchers from their staff. We informed them about the rationale of the study, the research question, and the data source. They were offered co-authorship of a subsequent publication of the study results and a payment of €3,000 for a full, well-documented analysis. Fifteen addressees agreed to participate, and 14 eventually took part in the study. Two further analyses were done independently by researchers of the Institute for Medical Informatics, Biometry and Epidemiology at the University Hospital in Essen (Germany). Thus, the study is based on analyses from 16 groups.

All analysts worked on the same research question: “Does marital status influence the incidence of cardiovascular disease (CVD)? Compare people who have never been married with married people who live with their partner“. The composite outcome was defined as “heart attack including myocardial infarction or coronary thrombosis or any other heart problem including congestive heart failure” or “stroke or cerebral vascular disease”. This question was intended to clearly specify exposure and outcome and to look for causality indicated by the verb “influence”. The word “incidence” suggested a longitudinal analysis. Analysts were expected to provide an effect estimate with 95% confidence interval (CI), a brief comment on their result, and the full syntax of their analysis. In addition to the main result, analysts were free to present results of further sensitivity analyses. Any statistical software could be used.

To answer the research question, all analysts were asked to use data from SHARE [20, 21]. SHARE data are freely available after registration, and they are documented in detail on the website of the study. To date, more than 4,000 publications have been published using SHARE data. SHARE was conducted to obtain data on health, social networks, and socioeconomic status of older people in 28 European countries and Israel. It is a panel study that started in 2004 and is harmonized between the bi-annual follow-up waves. To compensate for dropouts, new participants were recruited to the panel in waves 2, 4, 5, 6, and 7. The SHARE study was approved by the Ethics Committee of the University of Mannheim and the Ethics Council of the Max Planck Society. As wave 8 was not yet fully validated when the project started, analysts were asked to use only data from waves 1 to 7 of the survey, which were collected between 2004 and 2017. Wave 3 was not considered because it only contains data from childhood and adolescence. Where possible, all countries should be included in the analyses.

We created a forest plot showing the effect estimates with 95% CI for all 16 main analyses. Results were also presented separately for analyses strictly performed longitudinally and for analyses which were either strictly cross-sectional or which presented a mixed approach with elements of longitudinal and cross-sectional analyses. In interpreting the results, we described the following study characteristics: size of the analytical sample, inclusion criteria, definition of the exposure, definition of the outcome, handling of missing data, adjustment for confounding, choice of regression models, comparison of main and sensitivity analyses, as well as idiosyncratic decisions in the analyses.

To get an idea of how researcher degrees of freedom affect the effect estimates, one team selected exemplary regression models and performed the following own analyses in addition to the analyses of the 16 groups:

Additional analyses 1: To compare effect estimates from cross-sectional and longitudinal analyses, three additional regression analyses were conducted, two cross-sectional analyses of wave 1 and one longitudinal analysis with wave 1 as the baseline. The three analyses were identical with respect to the definition of exposure, outcome, confounders (age, sex, education) and the regression method used (Poisson regression with robust variance estimation). The first cross-sectional analysis included 22,984 individuals. In the second cross-sectional analysis, 5204 individuals were excluded for whom no follow-up data were available for the outcome. In the longitudinal analysis, further 2331 individuals who had the target event at wave 1 were missing. The smaller samples were therefore subsets of the larger samples.

Additional analyses 2: To study how strongly changes in the definitions of exposure and outcome affected the effect estimate, longitudinal analyses with wave 1 as baseline were performed holding all other factors constant (i.e., the same sample size, loglinear models with a Poisson working likelihood and robust standard errors, adjustment for age, sex, and education). Five definitions of the outcome were used: (1) use of the SHARE variables ph006d1 and ph006d4 (“Has a doctor ever told you that you had heart attack (…) / stroke (…)?”, (2) definition (1) or death by heart attack (SHARE variable xt011from end-of-live interviews in waves 5, 6 and 7), (3) definition of the outcome using the item whether participants had an event since the last interview (SHARE variables ph067_1 and ph067_2 in wave 2, and ph072_1 and ph072_2 in waves 4, 5, 6 and 7, respectively), (4) participants have the outcome if definitions (2) or (3) are fulfilled, (5) participants have the outcome if definitions (1) and (3) are fulfilled. Each of these outcome definitions was combined with two definitions of the exposure which was inclusion or exclusion of participants upon change of exposure status during follow-up according to SHARE variable dn044_.

Additional analyses 3: To study how strongly the choice of adjustment sets affected the effect estimate, longitudinal analyses (loglinear models with a Poisson working likelihood and robust standard errors) with wave 1 as baseline were conducted with all adjustment sets chosen by the participants. All other factors were held constant, in particular the size of the analysis dataset by using the imputed data provided by SHARE (*N* = 15,449).

## Results

Of the 16 groups of analysts, 12 consisted of only one person, two consisted of two people, one consisted of three, and one consisted of five people. Of these 24 persons, twelve were mathematicians / statisticians, six were epidemiologists, one was a medical doctor, one a physicist, one a psychologist, one a demographer, one an expert in survey methodology and one a sociologist. Six analysts had professorial titles, twelve had PhDs, and six were PhD students. All but one had experience as a first author of publications indexed in PubMed. The statistical software used for the project was R (nine times), SAS (five times), and STATA (two times).

**Fig. 1:**
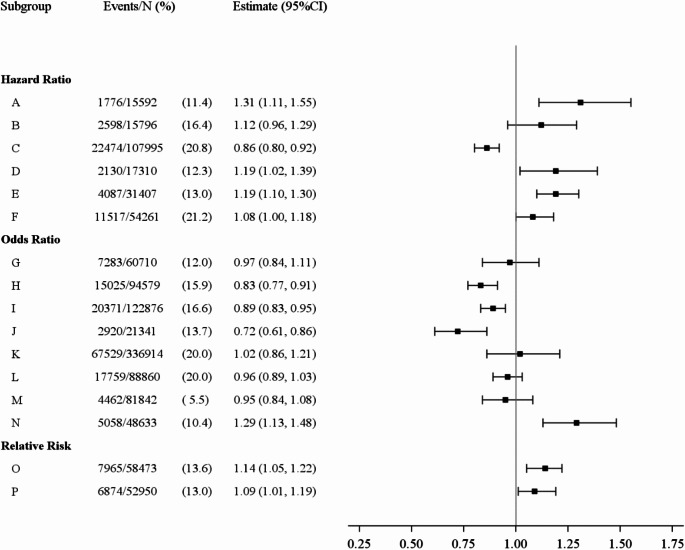
Effects estimates for the association between marital status (never married versus cohabiting married) and cardiovascular disease from the main analyses of the 16 analysts groups

**Fig. 2:**
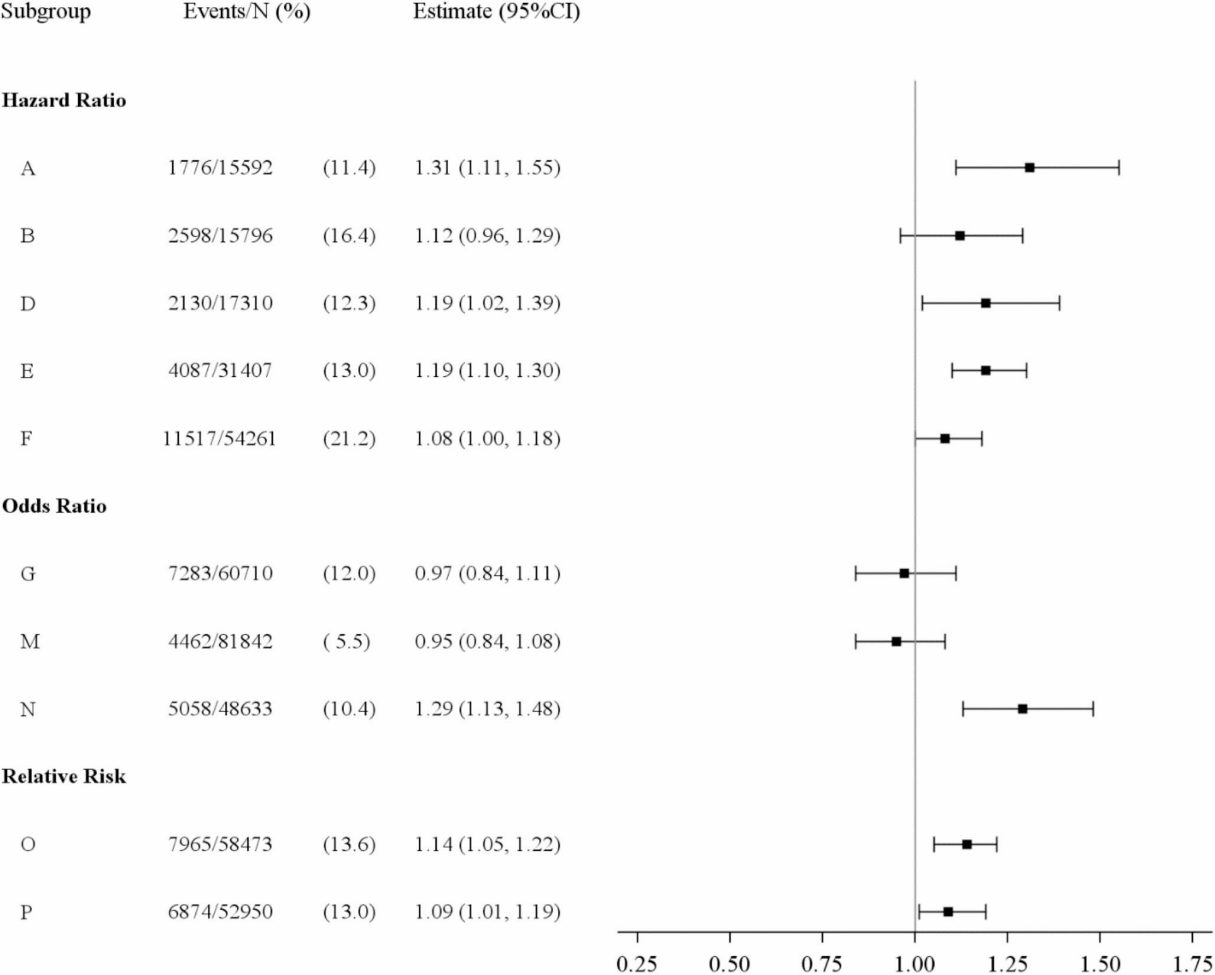
Effect estimates for the association between marital status (never married versus cohabiting married) and cardiovascular disease only from strictly longitudinal analyses

**Fig. 3:**
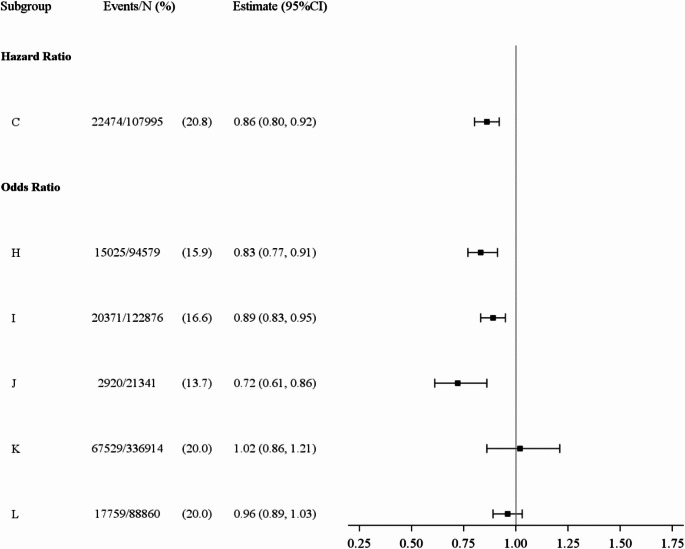
Effect estimates for the association between marital status (never married versus cohabiting married) and cardiovascular disease from strictly or partly cross-sectional analyses

Analysts estimated hazard ratios, odds ratios and relative risks ranging from 0.72 to 1.31 (reference: married and living together with partner) (Fig. 1). In the ten strictly longitudinal analyses, effect estimates ranged from 0.95 to 1.31 (Fig. 2). In the three longitudinal analyses with wave 1 as the only baseline, hazard ratios were 1.12, 1.19 and 1.31 (A, B, D in Fig. 2). In the six strictly or partly cross-sectional analyses, effect estimates ranged from 0.72 to 1.02 (Fig. 3).

Additional analyses 1 showed that the relative risks were 0.76 (95% CI: 0.66–0.88) for the first cross-sectional analysis (*N* = 22,984), 0.76 (95% CI: 0.64–0.91) for the second cross-sectional analysis (*N* = 17,780), and 1.10 (95% CI: 0.97–1.26) for the longitudinal analysis (*N* = 15,449).

The size of the 16 study populations ranged from 15,592 to 336,914 observations, and it depended strongly on whether the analysis was longitudinal or cross-sectional. The study samples were smallest in the three longitudinal analyses with wave 1 as the only baseline (range: 15,592 to 17,310 individuals). In the seven longitudinal analyses that additionally included SHARE participants newly recruited at waves 2, 4, 5 and 6, the size of the study population ranged from 31,407 to 81,842 subjects. The size of the study population was largest in an analysis with cross-sectional data from waves 1, 2, 4, 5, 6 and 7 (*n* = 336,914): in this analysis, many participants were included more than once, which was accounted for by using a random effect for the individual in the regression analysis. Exclusion criteria were often rather idiosyncratic, e.g.: exclusion of subjects married before the age of 18, exclusion of subjects from Israel, of persons under the age of 55 at recruitment, and subjects born after 1954. This has an impact on the size of the datasets. In wave 1, in 30,424 participants, 880 (2.9%) stated to have married under 18, 2449 (8.0%) were from Israel, 6633 (21.8%) were under 55 at recruitment, and 1194 (3.9%) were born after 1954.

Analysts dealt with the change in exposure across waves in different ways: in five analyses, SHARE participants with change in exposure were excluded; in five further analyses, this change was neglected; in two analyses, SHARE participants were censored after change in exposure; in three analyses, time-dependent exposure was used; in one cross-sectional analysis with data only from wave 1, change in exposure was not relevant. Furthermore, for two analysts, the exposure categories formed deviated from the predefined exposure categories “never married” and “cohabiting married”: one analyst added people with a registered civil partnership to those married and cohabiting, another combined cohabiting married, registered civil partnership, and married living apart.

To define the outcome variable, several variables were available in SHARE which could be considered: heart attack / stroke ever; age at heart attack / stroke; heart attack / stroke since last interview; main cause of death from end-of-life interviews with relatives of deceased participants in wave 7. The comparison of the 16 analyses revealed a large variability regarding the variable used to define the outcome. From the four variables available in SHARE for the outcome, only one was used in four analyses, two were used in four analyses, three in seven analyses, and all four in one analysis (Supplementary Table 1).

Table 1 shows the influence of different definitions of the exposure and the outcome on the effect estimates. For the ten variants used in the additional analyses 2, the relative risks ranged from 1.101 to 1.229.

**Table 1:** Relative risks with 95% confidence interval of longitudinal analyses (baseline = wave 1) dependent on the combination of five outcome definitions and two exposure definitions ^a, b^

Outcome ^c^	Inclusion of persons after change of exposure (*N* = 15,449)	Exclusion of persons after change of exposure (*N* = 13,102)
	RR (95% CI)	RR (95% CI)
CVD_1	1.102 (0.967–1.256)	1.119 (0.972–1.288)
CVD_2	1.134 (1.005–1.280)	1.151 (1.012–1.309)
CVD_3	1.126 (0.925–1.371)	1.221 (0.993–1.500)
CVD_4	1.148 (1.023–1.289)	1.167 (1.031–1.320)
CVD_5	1.101 (0.817–1.484)	1.229 (0.903–1.674)

^a^ The relative risks refer to the association between marital status (never married versus cohabiting married) and cardiovascular disease

^b^ The adjustment set (age, sex, education) and the regression model were identical in all 10 models

^c^ For the five outcome definitions cf. methods section (additional analyses 2)

All analysts performed complete case analyses, and no analyst used strategies such as multiple imputation. Three analysts occasionally used imputation data provided by SHARE. One group of analysts used “no information” as category for categorical variables to avoid exclusions.

The 16 groups of analysts provided 14 different adjustment sets for confounding (Table 2). Two did not adjust for confounding at all, two further analysts adjusted only for sex and age. The largest adjustment set included 11 variables. In two adjustment sets, the square root of age and age squared, respectively, were included in addition to age as a continuous variable. One analyst used restricted splines for education and age as timescale in the Cox regression model. Eight analysts did not give a rationale for their choice of adjustment variables. Three used directed acyclic graphs (DAGs), but the DAGs also varied considerably. Two groups referred to published papers, one used change in estimate, and one listened to gut feeling. One group explained that the research question was not aiming at causal inference in their views and did not adjust for confounders at all.

**Table 2:** Adjustment sets from the 16 analysts groups

Adjustment set	Frequency	Variables (Study group according to Fig. 1)
a	2	no confounders (C, F)
b	2	age, sex (H, J)
c	1	age, square root of age, sex (L)
d	1	age, sex, country (I)
e	1	age, sex, education (P)
f	1	age, sex, education, country (D)
g	1	age, age squared, sex, region (6 regions), smoking ever (O)
h	1	age, sex, education, income (tertiles), children (yes / no), country (G)
i	1	education (as restricted splines) (E)
j	1	age, sex, country, hypertension, lipid disorder, diabetes, smoking (B)
k	1	age, sex, country, hypertension, hypercholesterolaemia, diabetes, BMI, smoking, physical activity, depression, self-perceived health (A)
l	1	age, sex, BMI, smoking, physical activity, having a job, hospital stay in the last 12 months, depression (K)
m	1	age, sex, hypertension, increased value of cholesterol, physical activity, smoking, country, intake of drugs in cardiovascular drugs (N)
n	1	age, sex, BMI, chronic diseases, smoking, physical activity, country (M)

**Fig. 4:**
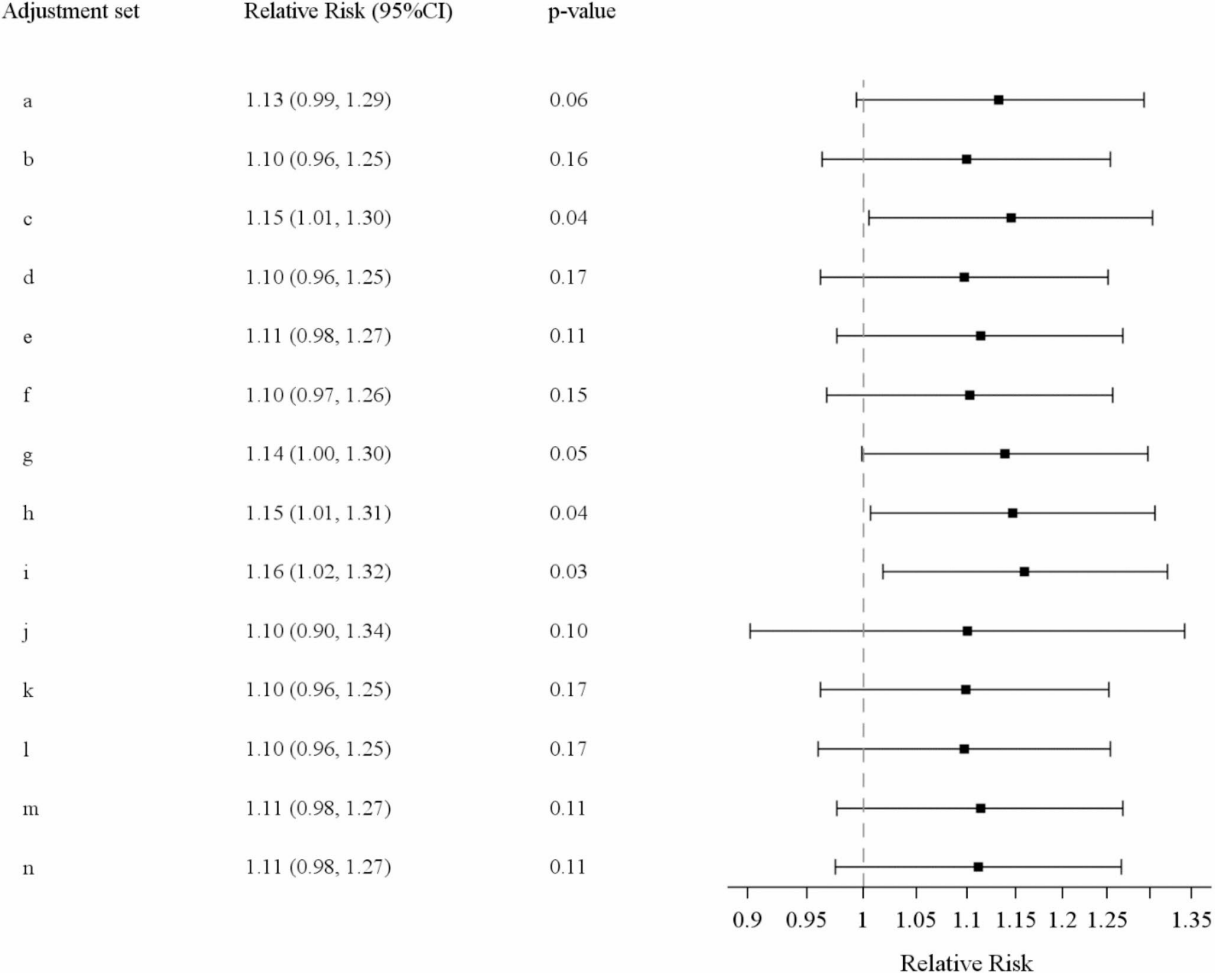
Relative risks with 95% confidence interval (CI) of a longitudinal analysis (baseline = wave 1) with all adjustment sets used by the 16 analysts groups (for adjustments sets, cf. Table 1)

In the longitudinal analyses with wave 1 as baseline (*N* = 15,449), the relative risks with the adjustment sets chosen by the participants ranged between 1.10 and 1.16 (additional analyses 3, Fig. 4).

Different regression models were used for the main result: logistic regression (five times), mixed-effects logistic regression (once), log-binomial model (twice), Cox Proportional Hazards model (six times), discrete time mixed effect model (once), and generalized estimating equations (once). Five groups performed sensitivity analyses using different regression models: except for one analyst using generalized estimating equations (GEE model) in addition to a Cox model, the choice of the regression model had little influence on the effect estimate (Table 3). E.g., in one analysis, the OR was 0.97 (95% 0.84–1.11) when a discrete time mixed model was used, and the HR was 0.99 (95% CI: 0.86–1.13) when a Cox regression model was fitted.

**Table 3:** Effect estimates with 95% confidence intervals from main and sensitivity analyses: results from 5 analysts groups

		Effect estimates ^a, b^ (95% CI)	*N*	Events
1	GEE	**OR = 0.89 (0.83–0.95)**	122,876	20,371
	Cox regression	HR = 1.18 (0.91–1.52)	93,333	1,262
2	Logistic regression	OR = 1.32 (1.10–1.59)		
	Cox regression	**HR = 1.31 (1.11–1.55)**	15,592	1,776
3	Discrete time mixed effect model	**OR = 0.97 (0.84–1.11)**	60,710	7,283
	Cox regression	HR = 0.99 (0.86–1.13)		
4	Nested Case Control Study	OR = 1.06 (0.97–1.17)		
	Cox Regression	**HR = 1.08 (1.00–1.18)**	54,261	11,517
5	Logistic regression	**OR = 1.29 (1.13–1.48)**	48,633	5,058
	Cox regression	HR = 1.25 (1.10–1.41)		

GEE: generalized estimated equations; OR: odds ratio; HR: hazard ratio; CI: confidence interval

^a^ The effect estimates refer to the association between marital status (never married versus cohabiting married) and cardiovascular disease

^b^ The effect estimate of the main analysis is highlighted in bold

In analyses using Cox models, calculations of survival time differed. One group of analysts used the number of waves as a proxy of time. One used the time between baseline and last wave if there was no event, and time between baseline and penultimate wave plus half time between penultimate and last wave if an event occurred. Two used the time between baseline and last wave regardless of whether an event took place or not. Four used age as a proxy for time on study. None of the groups checked the proportional hazards assumption. All analysts but one used the default for ties which is Breslow in SAS and STATA, and Efron in R.

Some groups criticized the quality of SHARE data. One group showed that in the analysis data-set age at diagnosis of outcome and age at marriage were missing for about 50% of subjects. Three other groups pointed out that self-reports of outcomes and marital status were often inconsistent.

## Discussion

In this multi-analyst study, each analysis was unique. There were no two analyses that were even close to similar, and the size of the analysis data sets varied considerably. The effect estimates (hazard ratios, odds ratios, or relative risks) for the association between marital status (cohabiting married versus never married) and cardiovascular events ranged from 0.72 to 1.31. The variation in effect estimates was smaller when the strictly longitudinal studies (and longitudinal analyses with wave 1 as the only baseline in particular) and the strictly or partly cross-sectional studies were considered separately. Almost each group of analysts used a different adjustment set for confounding, which had only a small effect on the range of effect estimates in our example data set (relative risks ranged from 1.10 to 1.16). Differences in the precise definition of exposure and outcome, and the choice of the regression models also had an only small influence on the effect estimate. Modern epidemiological and statistical methods such as multiple imputation, directed acyclic graphs, target trial emulation, and inverse probability treatment weighting were rarely or never used. Only three groups used imputation data provided by SHARE for single variables.

Effect estimates for the association between marital status and cardiovascular outcomes from strictly longitudinal analyses in the present multi-analyst study were comparable to effect estimates from previous studies, although closer to the null effect. A meta-analysis with 34 prospective studies showed associations between marital status (unmarried versus married) and cardiovascular events (OR = 1.42 (95% CI: 1.00–2.01)), stroke events (OR = 1.23 (0.93–1.63)) and stroke deaths (OR = 1.55 (1.16–2.08)) [22]. Other recent studies have shown similar results [23, 24].

An interpretation of the results of the 16 study groups as widely different may be inappropriate. The sensitivity analyses by some analysis groups and the additional analyses 2 and 3 suggest that researcher degrees of freedom like the choice of precise definitions of exposure and outcome, the choice of adjustment sets, and the choice of regression models each had only small impact on the effect estimates. Cross-sectional analyses, longitudinal analyses with only wave 1 as baseline, and longitudinal analyses including also participants recruited at later waves actually answer different questions. The sizes of the analysis data sets differed strongly according to the different study types. Results of analyses with comparable research questions differed less. For longitudinal analyses with wave 1 as the only baseline, the three hazard ratios were 1.12, 1.19 and 1.31. Among the cross-sectional analyses, analysis J which is a strictly cross-sectional analysis of wave 1 and analysis K which is a strictly cross-sectional analysis of all waves and which includes many participants more than once, are barely comparable to the remaining four partly cross-sectional analyses for which effect estimates range from 0.83 to 0.96. The different results from cross-sectional and longitudinal analyses may be explained by the prevalence incidence bias which refers to the influence of the exposure on survival after the outcome. In particular, never-married men are more likely to die after a heart attack or stroke than married men and are therefore less likely to be included in a cross-sectional study after the event [25–27]. Moreover, in a cross-sectional study at wave 1, cases before wave 1 are considered, while in a longitudinal study starting at wave 1, incident cases between wave 1 and wave 7 are considered. The additional analysis 1 clearly demonstrates that the different results from cross-sectional and longitudinal analyses do not result from confounding by other factors like definition of exposure and outcome, the choice of the adjustment set or the choice of the regression model. It may be surprising that not all groups have carried out longitudinal analyses using data from a panel study. One explanation may be the rather complicated study design of SHARE, in particular new countries joining the study in later waves and refreshment samples from wave 2 onwards combined with the perception, that the task should be managed in a defined time.

A common observation of most previous multi-analyst studies was a wide range of results - from positive to negative effects. There were only few exceptions: in a study by Hoogeven et al. with 120 teams of analysts, all but three reported a statistically significant correlation between religiosity and well-being [14]. In a study by Veronese et al. 14 teams reported “similar although not identical results” despite different evaluation strategies [9]. Van Dongen et al. gave two deliberately very simple data sets (2 × 2 table; 13 value pairs (perceived stress, activity of the amygdala)) to four teams of very experienced statisticians [28]. Despite very different methodical approaches (e.g. two times frequentists, two times Bayesian) the authors drew the same conclusion that the data were inconclusive for both research questions analyzed in that study.

For the mixed results in earlier multi-analysist studies, different reasons were given. Breznau et al. stated that 95% of the variability of the results were due to non-identifiable decisions in the analyses and called this “idiosyncratic variation” [12]. Sociologists conducted a re-analysis of the football study by Silberzahn et al. [11, 29]. They identified four different interpretations of the research question of that study and attributed the variance in results to a lack of clarity in the research question [29]. Others showed that peer confidence, a proxy for expertise, was strongly related to the variability of results in the study by Silberzahn: the higher the peer confidence, the more similar the results [30]. In another multi-analyst study in neuroscience, different correction methods for multiple testing and different software were identified as reasons for mixed results [8]. Traditions in institutions may also play a role: in the present study, the only two analysts who used log-binomial regression models to estimate relative risks worked at the same institute. We assume that there was no incentive for bias or p-hacking in this study: First, the analysts had not chosen the topic and therefore had no preference for a specific outcome. Second, they knew that the chances of future co-authorship did not depend on whether their results were positive, negative, or neutral.

Several suggestions have been made to deal with researcher degrees of freedom, including making the data and syntax freely available, and conducting multiverse and multi-analyst studies [1, 31, 32]. Providing the syntax of the statistical analyses in a supplement can increase transparency. However, it is very unlikely that many readers of a paper will read the syntax line by line because this takes time and readers may not be familiar with the statistical software used. Changing the syntax in terms of micro-decisions may lead to changes in the results that would not justify a second publication in a scientific journal. In multiverse analyses, researchers perform a large variety of own analyses and present them all in one publication. For example, Levitt et al. estimated 66 excess mortalities during the SARS-CoV-2 pandemic by using 66 different reference periods rather than reporting the result for a single subjectively chosen reference period [33]. Wagenmakers et al. suggested that more multi-analyst studies should be conducted, and they recommended that journals create formats and incentives for this, e.g., presentation of alternative analyses in the supplement, either by themselves or by other analysts, or comments on accepted articles in which other authors present their own analyses of the same data set [19].

In the present study, much of the variation can be attributed to the type of study on which the analyses were based (cross-sectional, longitudinal, hybrid), and to the correspondingly large differences in the size of the analysis data sets. The reason for these different approaches may either be that the research question still left some non-intended room for interpretation. Or the characteristics of the SHARE dataset let some analysts prefer a (partly) cross-sectional analysis which is easier to perform. Beyond this, it should be noted that there were also differences in the quality of the analyses. Longitudinal analyses should be preferred to cross-sectional studies, analyses with multiple imputation should be preferred to analyses without imputation, and analyses that take into account the time dependence of exposure are formally preferable to analyses that do not take this into account. From those aspects, only the longitudinal versus cross-sectional comparison could be studied empirically in the current study.

In other fields, particularly in psychology, there is lively discussion on lack of reproducibility of studies. In our view, the discussion of reproducibility in epidemiology has not been as strong as in other fields. This may be explained by the fact that reproducibility is mainly inferential reproducibility, and thus based on dichotomization of p-values. In epidemiology, there is a trend away from orthodox null hypothesis significance testing towards the estimation of point estimates with 95% confidence intervals [34–36]. Therefore, in our multi-analysts study we were mainly interested in the variability of point estimates. Our results show that many “classical” aspects of epidemiology (type of regression model, definition of outcome or exposure, selection of confounders) played only a minor role, in contrast to the choice between cross-sectional and longitudinal analysis.

Rather, it seems that not all analyses gave answers to the same research questions, i.e. that there were different “estimands”. An estimand is “.the target of estimation to address the scientific question of interest posed by the trial objective” and has five attributes – population, treatment, variable (endpoint), intercurrent events and the summary measure – that must be well defined before analysis [37, 38]. For example, we could be interested in the effect of the one-time treatment “getting married” in terms of “tying the knot” or “exchanging vows” or in the cumulative effect over time of the treatment “marriage” involving shared responsibilities, rights, and benefits. Such a time-varying treatment cannot be investigated in the cross-sectional setting. The target population can either be anyone who could get married (or has already married) or anyone who has not experienced the outcome yet at the time of recruitment. The latter would need longitudinal analyses that refer to incident cases of the outcome after baseline whereas the former could also be investigated in cross-sectional analyses that sum up cases given they survived until the time of measurement. Another distinction could be between the legal perspective (being married vs. not) on the one side and social binding (living together without marriage) and this in various cultural contexts.

Our study has some limitations. First, the number of analysts was small. Second, there was little heterogeneity in the experience of the analysts, so the influence of the level of experience on the results could not be investigated. Third, we only included researchers in Germany, except for one group from Switzerland and Denmark each. Researchers from other countries may have used other techniques due to country specific trends and teaching topics at universities. Fourth, having the same effect estimate from all analyses would have been favorable for comparability. However, we did not restrict the choice of regression models because this is an important degree of freedom in data analysis. Therefore, we accepted different summary measures (e.g. odds ratios and hazard ratios) in the analyses. Nevertheless, as the disease risk was not very high (i.e. < 20%) the deviation between the figures for the different effect estimates may not be large. Fifth, we did not analyze the association between prior beliefs on the research question and results of the analysis. Finally, the research question was not implemented as intended by those analysts who did not perform a strictly longitudinal analysis. The explanation may be that these analysts did not interpret the research question as intended, or that they preferred (partly) cross-sectional analyses because of the complex structure of SHARE data and because age at diagnosis of outcome and age at marriage were missing for many subjects.

A strength of our study is that we used data from SHARE, the largest and very well curated European social science panel study, which is the basis of 4,119 publications up to October 2024. As mentioned above, some analysts considered data quality as poor at least for some variables. In the context of a multi-analyst study, this may be even a strength because it means that the analysts of our study were confronted with problems that are not uncommon in the analysis of epidemiological data.

## Conclusion

The present multi-analyst study with an epidemiological research question gives a less pessimistic view than similar studies from other scientific disciplines. At first sight, there is a wide range of results especially in analyses of purely longitudinal and cross-sectional type which give answers to different research questions. However, analyses with similar estimands and similar sample sizes gave more homogeneous results. The additional analyses on the effect of researcher degrees of freedom (the precise definition of exposure and outcome, choice of the adjustment set, choice of the regression model) suggest that these decisions had only a small influence on the effect estimates.

In our study, the researcher degrees of freedom were arbitrary choices made by the analysts. To see how strongly effect estimates depend on researcher degrees of freedom, a systematic multiverse analysis that takes into account all possible analytical choices would be ideal. However, in a study with a rather complex data set like SHARE, this is hardly feasible. For future research, we suggest conducting multiverse analyses for small and manageable datasets. The research question had used the verb “influence”, which is causal language and the word “incidence”, and those who developed the question imagined that all analysts would perform longitudinal analyses and include participants recruited not only at wave 1, but also at later waves. For future multi-analyst studies, we suggest that the research question be formulated even more rigorously.

With our study, we aimed to initiate the discussion on subjective, but legitimate decisions in data analysis in epidemiology, and we look forward to further studies on this topic.

## Electronic supplementary material

Below is the link to the electronic supplementary material.

Supplementary Material 1

## References

[1] Hoffmann S, Schönbrodt F, Elsas R, Wilson R, Strasser U, Boulesteix AL. The multiplicity of analysis strategies jeopardizes replicability: lessons learned across disciplines. R Soc Open Sci. 2021;8:201925. 10.1098/rsos.201925. 33996122 10.1098/rsos.201925 PMC8059606

[2] Patel CJ, Burford B, Ioannidis JPA. Assessment of vibration of effects due to model specification can demonstrate the instability of observational associations. J Clin Epidemiol. 2015;68:1046–58. 10.1016/j.jclinepi.2015.05.029. 26279400 10.1016/j.jclinepi.2015.05.029 PMC4555355

[3] Steegen S, Tuerlinckx F, Gelman A, Vanpaemel W. Increasing transparency through a multiverse analysis. Perspect Psychol Sci. 2016;11:702–12. htps://. 27694465 10.1177/1745691616658637

[4] Simonsohn U, Simmons JP, Nelson LD. assessed October 18,. Specification curve: descriptive and inferential statistics on all reasonable specifications. Available at SSRN: https://papers.ssrn.com/sol3/papers.cfm?abstract_id=2694998 (2024).

[5] Menkveld AJ, Dreber A, Holzmeister F et al. assessed July 23,. Non-Standard Errors. Journal of Finance Forthcoming, available at SSRN: https://papers.ssrn.com/sol3/papers.cfm?abstract_id=3961574 (2023).

[6] Huntington-Klein N, Arenas A, Beam E, et al. The influence of hidden researcher decisions in microeconomics. Econ Inq. 2021;59:944–60. 10.1111/ecin.12992.

[7] Schweinsberg M, Feldman M, Staub N, et al. Same data, different conclusions: radical dispersion in empirical results when independent analysts operationalize and test the same hypothesis. Organ Behav Hum Decis Process. 2021;165:228–49. 10.1016/j.obhdp.2021.02.003.

[8] Botvinik-Nezer R, Holzmeister F, Camerer CF, Dreber A, Huber J, Johannesson M, et al. Variability in the analysis of a single neuroimaging dataset by many teams. Nature. 2020;582:84–8. 10.1038/s41586-020-2314-9. 32483374 10.1038/s41586-020-2314-9 PMC7771346

[9] Veronese M, Rizzo G, Belzunce M, et al. Reproducibility of findings in modern PET neuroimaging: insight from the NRM2018 grand challenge. J Cereb Blood Flow Metab. 2021;41:2778–96. 10.1177/0271678X211015101. 33993794 10.1177/0271678X211015101 PMC8504414

[10] Fillard P, Descoteaux M, Goh A, et al. Quantitative evaluation of 10 tractography algorithms on a realistic diffusion MR Phantom. NeuroImage. 2011;56:220–34. 10.1016/j.neuroimage.2011.01.032. 21256221 10.1016/j.neuroimage.2011.01.032

[11] Silberzahn R, Uhlmann EL, Martin DP, et al. Many analysts, one data set: making transparent how variations in analytic choices affect results. Adv Methods Pract Psychol Sci. 2018;1:337–56. 10.1177/2515245917747646.

[12] Breznau N, Rinke EM, Wuttke A, et al. Observing many researchers using the same data and hypothesis reveals a hidden universe of uncertainty. PNAS. 2022;119:e2203150119. 10.1073/pnas.2203150119. 36306328 10.1073/pnas.2203150119 PMC9636921

[13] Salganik MJ, Lundberg I, Kindel AT, et al. Measuring the predictability of life outcomes with a scientific mass collaboration. PNAS. 2020;117:8398–403. 10.1073/pnas.2118703118. 32229555 10.1073/pnas.1915006117 PMC7165437

[14] Hoogeveen S, Sarafoglou A, Aczel B, et al. A many-analysists approach to the relation between religion and well-being. Relig Brain Behav. 2022. 10.1080/2153599X.2022.2070255.

[15] Starns JJ, Cataldo AM, Rotello CM, et al. Assessing theoretical conclusions with blinded inference to investigate a potential inference crisis. Adv Methods Pract Psychol Sci. 2019;2:335–49. 10.1177/2515245919869583.

[16] Boehm U, Annis J, Frank MJ, et al. Estimating across-trial variability parameters of the diffusion decision model: expert advice and recommendations. J Math Psychol. 2018;87:46–75. 10.1016/j.jmp.2018.09.004.

[17] Dutilh G, Annis J, Brown SD, et al. The quality of response time data inference: a blinded, collaborative assessment of the validity of cognitive models. Psychon Bull Rev. 2019;26:1051–69. 10.3758/s13423-017-1417-2. 29450793 10.3758/s13423-017-1417-2 PMC6449220

[18] Aczel B, Szaszi B, Nilsonne G, et al. Consensus-based guidance for conducting and reporting multi-analyst studies. eLife. 2021;10:e72185. 10.7554/eLife.72185. 34751133 10.7554/eLife.72185 PMC8626083

[19] Wagenmakers EJ, Sarafoglou A, Aczel B. One statistical analysis must not rule them all. Nature. 2022;605:423–5. 10.1038/d41586-022-01332-8. 35581494 10.1038/d41586-022-01332-8

[20] Börsch-Supan A, Brandt M, Hunkler C, et al. Data resource profile: the survey of health, ageing and retirement in Europe (SHARE). Int J Epidemiol. 2013;42:992–1001. 10.1093/ije/dyt088. 23778574 10.1093/ije/dyt088 PMC3780997

[21] SHARE -. Survey of Health, Ageing and Retirement in Europe. https://share-eric.eu

[22] Wong CW, Kwok CS, Narain A, et al. Marital status and risk of cardiovascular diseases: a systematic review and meta-analysis. Heart. 2018;104:1937–48. 10.1136/heartjnl-2018-313005corr1. 29921571 10.1136/heartjnl-2018-313005

[23] Wang Y, Jiao Y, Nie J, et al. Sex differences in the association between marital status and the risk of cardiovascular, cancer, and all-cause mortality: a systematic review and meta-analysis of 7,881,040 individuals. Glob Health Res Policy. 2020;5:4. 10.1186/s41256-020-00133-8. 32161813 10.1186/s41256-020-00133-8 PMC7047380

[24] Leung CY, Huang HL, Abe SK, et al. Association of marital status with total and cause-specific mortality in Asia. JAMA Netw Open. 2022. 10.1001/jamanetworkopen.2022.14181. 35639382 10.1001/jamanetworkopen.2022.14181 PMC9157263

[25] Schmaltz HN, Southern D, Ghali WA, Jelinski SE, Parsons GA, King KM, et al. Living alone, patient sex and mortality after acute myocardial infarction. J Gen Intern Med. 2007;22(5):572–8. 10.1007/s11606-007-0106-7. 17443363 10.1007/s11606-007-0106-7 PMC1852915

[26] Kilpi F, Konttinen H, Silventoinen K, Martikainen P. Living arrangements as determinants of myocardial infarction incidence and survival: A prospective register study of over 300,000 Finnish men and women. Soc Sci Med. 2015;133:93–100. 10.1016/j.socscimed.2015.03.054. 25863724 10.1016/j.socscimed.2015.03.054

[27] Redfors P, Isaksén D, Lappas G, et al. Living alone predicts mortality in patients with ischemic stroke before 70 years of age: a long-term prospective follow-up study. BMC Neurol. 2016;16:80. 10.1186/s12883-016-0599-y. 27411309 10.1186/s12883-016-0599-y PMC4942912

[28] Van Dongen NNN, van Doorn JB, Gronau QF, et al. Multiple perspectives on inference for two simple statistical scenarios. Am Stat. 2019;73:328–39. 10.1080/00031305.2019.1565553.

[29] Auspurg K, Brüderl J. Has the credibility of the social sciences been credibly destroyed? Reanalyzing the many analysts, one data set. Project Socius. 2021;7. 10.1177/23780231211024421.

[30] Kummerfeld E, Jones GL. One data set, many analysts: implications for practicing scientists. Front Psychol. 2023;14:1094150. 10.3389/fpsyg.2023.1094150. 36865366 10.3389/fpsyg.2023.1094150 PMC9971968

[31] Wagenmakers EJ, Sarafoglou A, Aarts S, et al. Seven steps toward more transparency in statistical practice. Nat Hum Behav. 2021;5:1473–80. 10.1038/s41562-021-01211-8. 34764461 10.1038/s41562-021-01211-8

[32] Olsson-Collentine A, van Aert RCM, Bakker M, Wicherts J. Meta-analyzing the multiverse: a peek under the Hood of selective reporting. Psychol Methods. 2023. 10.1037/met0000559. 37166859 10.1037/met0000559

[33] Levitt M, Zonta F, Ioannidis JPA. Excess deaths estimates from multiverse analyses in 2009–2021. Eur J Epidemiol. 2023;38:1129–39. 10.1007/s10654-023-00998-2. 37043153 10.1007/s10654-023-00998-2 PMC10090741

[34] Stang A, Poole C, Kuss O. The ongoing tyranny of statistical significance testing in biomedical research. Eur J Epidemiol. 2010;25:225–30. 10.1007/s10654-010-9440-x. 20339903 10.1007/s10654-010-9440-x

[35] Amrhein V, Korner-Nievergelt F, Roth T. The Earth is flat (p > 0.05): significance thresholds and the crisis of unreplicable research. PeerJ. 2017;5:e3544. 10.7717/peerj.3544. 28698825 10.7717/peerj.3544 PMC5502092

[36] Amrhein V, Greenland S, McShane B. Scientists rise up against statistical significance. Nature; 567(7748):305–7. 10.1038/d41586-019-00857-9 10.1038/d41586-019-00857-9 30894741

[37] Lawrance R, Degtyarev E, Griffiths P, et al. What is an estimand & how does it relate to quantifying the effect of treatment on patient-reported quality of life outcomes in clinical trials? J Patient Rep Outcomes. 2020;4:68. 10.1186/s41687-020-00218-5. 32833083 10.1186/s41687-020-00218-5 PMC7445213

[38] Casey M, Degtyarev E, Lechuga MJ, et al. Estimand framework: are we asking the right questions? A case study in the solid tumor setting. Pharm Stat. 2021;20:324–34. 10.1002/pst.2079. 33155417 10.1002/pst.2079

