# Supplementary material for: Marital status and risk of cardiovascular disease – a multi-analyst study in epidemiology
Source: Eur J Epidemiol. 2025 May 5;40(5):497–509. doi: 10.1007/s10654-025-01235-8 (PMC12170710; doi:10.1007/s10654-025-01235-8)
Supplement: Supplementary file 1 — Supplementary Material 1 [file 10654_2025_1235_MOESM1_ESM.docx]

**Supplementary table 1.** Variables used to define the outcome

|  | Variables related to the outcome | | | |
| --- | --- | --- | --- | --- |
| Analyst group | Ph006d1, ph006d4:  Heart attack / stroke ever | Ph009_1, ph009_4:  Age at heart attack / stroke | Ph067_1, ph067_2 / ph072_1, ph072_2:  Heart attack / stroke since last interview | Xt011_: Main cause of death heart attack / stroke |
| A | X (in wave 1) | X | X | X |
| B | X |  |  |  |
| C | X | X |  |  |
| D | X |  |  |  |
| E | X | X |  | X |
| F | X | X |  | X |
| G | X |  |  |  |
| H | X | X | X |  |
| I | X | X |  | X |
| J | X | X |  |  |
| K | X |  |  |  |
| L | X | X | X |  |
| M | X | X | X (and ph068_1; ph068_2; ph069_1; ph069_2) |  |
| N | X |  | X | X |
| O | X |  |  | X |
| P | X |  |  | X |
